# The second virial coefficient as a predictor of protein aggregation propensity: A self-interaction chromatography study

**DOI:** 10.1016/j.ejpb.2015.07.025

**Published:** 2015-10

**Authors:** A. Quigley, D.R. Williams

**Affiliations:** Surfaces and Particle Engineering Laboratory, Department of Chemical Engineering, Imperial College London, London SW7 2BY, UK

**Keywords:** Second virial coefficient, *b*_2_, Protein aggregation, Protein–protein interactions, Self-interaction chromatography, Lysozyme, Lactoferrin, Catalase, Concanavalin A, Protein stability

## Abstract

The second osmotic virial coefficients (*b*_2_) of four proteins – lysozyme, recombinant human lactoferrin, concanavalin A and catalase were measured by self-interaction chromatography (SIC) in solutions of varying salt type, concentration and pH. Protein aggregate sizes based on the initial hydrodynamic radius of the protein solution species present were measured using dynamic light scattering, and the relationship between *b*_2_ and protein aggregate size was studied. A linear correlation was established between *b*_2_ values and protein aggregate hydrodynamic size for all proteins, and for almost all solution conditions. Aggregate sizes of <∼10 nm, indicative of non-aggregated protein systems, were consistently observed to have *b*_2_ values >0. The observed *b*_2_ trends as a function of solution conditions were very much protein dependent, with notable trends including the existence of attractive interactions (negative *b*_2_ values) at low ionic strengths for catalase and concanavalin A, and the highly positive *b*_2_ values observed for lactoferrin over a wide range of solution conditions, reflecting lactoferrin’s innately high stability. It is concluded that the quantification of protein–protein interactions using SIC based *b*_2_ data is a potentially valuable screening tool for predicting protein aggregation propensity.

## Graphical abstract

**Figure:**
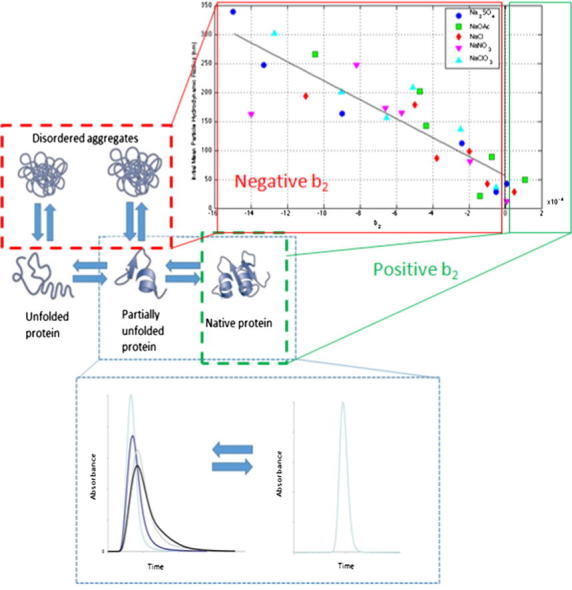

## 1 Introduction

Protein aggregation is known to occur at every stage in the production, formulation, storage, shipping and even during administration of protein-based therapeutics. As such protein aggregation is a problem of significant magnitude for the biopharmaceutical industry, and despite enormous technical advances in recent years it continues to be a major obstacle to development [16]. Therefore, the ability to predict, minimise, restrict and/or reverse protein aggregation is crucial to the viable manufacture and formulation of biotherapeutics. Unfortunately the control of aggregation is a considerable challenge because the mechanisms of aggregation follow numerous pathways, and although much knowledge of aggregation mechanisms has been accumulated it is still not currently possible to robustly predict a protein’s propensity to aggregate [46]. However, the current models of aggregation have identified two factors that govern stability; one is colloidal and the other is conformational. Colloidal stability is determined by the balance of repulsive and attractive intermolecular interactions between protein molecules in solution. Conformational stability is defined as the difference in free energy between the folded and unfolded states of a protein molecule. Current techniques for predicting protein aggregation propensity are therefore based on the assessment of conformational and colloidal stabilities. These include *in silico* sequence/structure based predictions [11] and determination of melting temperature (*T_m_*) as indicators of conformational stability [35] and the determination of the osmotic second virial coefficient (*B*_22_) as a measure of colloidal stability [47], [10].

*B*_22_ can be determined experimentally using static light scattering (SLS) [63], self-interaction chromatography (SIC) [59], membrane osmometry (MO) [32] and analytical ultracentrifugation (AUC) [3]. SIC has established itself as an important experimental technique for the measurement of the *B*_22_ with comparable results and several advantages over the more established SLS methodology, including reduced amounts of sample and shorter experimental times. *B*_22_ quantifies the magnitude and direction of protein–protein interactions in dilute solution. Measurement of *B*_22_ values has been identified as a method of great potential that could have a significant role in the prediction of protein aggregation where attractive protein–protein colloidal interactions are dominant. Negative *B*_22_ values denote net attractive protein–protein interactions whilst positive values represent overall repulsive interactions.

The use of *B*_22_ data as a semi-quantitative tool for predicting optimal solution conditions for crystallisation is now a well-documented approach [22], [23], [63], [7], [58], [19]. Of course a precursor stage, and indeed crucial step, to the crystal growth process is the formation of critical nuclei in solution. Such nucleation events are intrinsically related to aggregation so it is not surprising that *B*_22_ data could potentially be a useful screening tool for predicting protein aggregation propensity. A number of authors have reported on the potential utility of *B*_22_ as a predictor of protein aggregation propensity. Published work in this area has shown that protein aggregation behaviour is frequently well correlated to *B*_22_ values determined under the same conditions [60], [29], [13]. As such, screening for positive *B*_22_ values could be used for a rapid determination of high stability solution conditions for proteins.

It is perhaps unsurprising that *B*_22_ does not always reflect aggregation rates and propensities given that proteins probably belong to the most complex colloidal systems encountered, considering the possible variations in size, morphology-structure, surface charge and surface chemistry. A paper on the pH dependence of *B*_22_ and aggregation propensity of 3 monoclonal antibodies (mAbs) [49] reported that the correlation between aggregation propensities and *B*_22_ became insignificant when *B*_22_ values were negative, as such, even if the *B*_22_ values were the same the three mAbs exhibited different aggregation propensities. It should also be noted that conformational stability plays an important role in aggregation propensity, with partially unfolded conformational intermediates often considered as the main cause of aggregate formation. When a conformational change is responsible for the onset of aggregation, *B*_22_ does not always correlate with measured rates of aggregation [12]. Furthermore, a study on ovalbumin and a mAb conducted by Bajaj and co-workers [6], concluded that it was unlikely *B*_22_ would correlate with long term aggregation because the aggregation-prone structurally perturbed state could be present in a small fraction compared to the native species, yet the structural changes could be significant enough to lead to aggregation in the long term.

The present study investigates the relationship between the *B*_22_ and aggregation to gain a better understanding of the potential utility of this parameter to predict the propensity of a protein to undergo aggregation. Four different model proteins were used in this study; lysozyme (pI = 11.0, *M_W_* 14.3 kDa), catalase (pI 5.4, *M_W_* 250 kD), concanavalin A (con A) (pI 4.5–5.5, *M_W_* 104–112 kDa), and recombinant human lactoferrin (lactoferrin) (pI 9.5, *M_W_* 82 kDa). Instead of reporting *B*_22_ values which are dependent on the molecular weight of the protein, it is more appropriate for the comparison of different proteins that data are presented as the reduced or dimensionless osmotic second virial coefficient (*b*_2_) for which *b*_2_ is normalised by the excluded volume contribution $B_{2}^{\mathrm{HS}}$. *B*_2_ can easily be converted to *B*_22_ through the following equation [8]:

(1) $$b_{2}=\frac{B_{2}}{B_{2}^{\mathrm{HS}}}=\frac{3B_{2}}{2\pi \sigma^{3}}=\frac{B_{22}M_{w}^{2}}{N_{a}B_{2}^{\mathrm{HS}}}$$

All proteins studied were subjected to solution conditions intended to rapidly induce aggregation as well as those in which they were stable. *b*_2_ values were measured under similar conditions using the improved (first moment) method to determine retention times from SIC data recently reported [42]. It has been recently shown that *b*_2_ values obtained in this manner show more accurate correlation with protein aggregation and that peak shape may be itself an important indicator for conformational changes in protein samples. These observations regarding peak shape complement earlier work discussing the possibility that retention peak data contain information not only on the average *B*_22_ values typically reported, but for a range of *B*_22_ values reflecting the heterogeneity of protein solution interactions [42].

## 2 Materials and methods

### 2.1 Materials

Chicken egg white lysozyme (62971), catalase from bovine liver (C9322) and concanavalin A (con A) from *Canavaliaensiformis* (L7647) were purchased from Sigma Aldrich. Recombinant human lactoferrin (lactoferrin) was produced at Fujifilm Diosynth using *Aspergillus niger* as the expression system and purified by cation exchange chromatography. The concentration of lactoferrin was 100.7 mg/mL in phosphate buffered saline solution pH 7.5. Potassium phosphate, sodium cyanoborohydride, dibasic sodium phosphate, MES, *N*-(3-dimethylaminopropyl)-*N*′-ethylcarbodiimide-hydrochloride (EDC), *N*-hydroxysuccinimide (NHS), ethanolamine, HCl and NaOH were all purchased from Sigma–Aldrich (ACS or BioXtra grade). NaCl, Sodium acetate trihydrate, glacial acetic acid and acetone were purchased from Fisher Scientific and were AR grade. Toyopearl AF-Formyl-650M and AF-Amino-650M chromatography particles (08004 and 08002) were obtained from Tosoh Biosep. Deionised water used for preparing all buffer and protein solutions was processed by a Centra ELGA system. The pH was measured using a Mettler Toledo FiveEasy pH meter. All solutions were filtered prior to use using 0.22 μm filters from Millipore. Protein concentrations were determined by BCA protein assay using a kit obtained from Pierce and a Lambda 4B spectrophotometer from Perkin–Elmer.

### 2.2 Protein immobilisation

The immobilisation of lysozyme, lactoferrin and con A to Toyopearl AF-Formyl-650M particles was based on the method by Tessier et al. [59] as detailed here [42]. Catalase was immobilised to Toyopearl AF-Amino-650M particles using a method described by Dumetz et al. [18]. Between 65 mg and 110 mg of each protein were dissolved in 10 mL buffer solution (lysozyme in 0.1 M potassium phosphate at pH 7.5, lactoferrin in 20 mM sodium phosphate pH 7, catalase in 5 mM MES pH 6.5 containing 0.1 M NaCl, and con A in 20 mM sodium acetate pH 4.5). The coupling was catalysed using sodium cyanoborohydride for lysozyme, lactoferrin and con A and with a mixture of EDC and NHS for catalase. Any remaining active sites on the media were capped using ethanolamine. The protein loaded stationary phase was then slurry packed (at a flow rate of no more than 3 mL/min) into the column and washed *in situ*. Samples were collected from the initial protein solution and each of the washes in order to calculate the net amount of protein immobilised on the stationary phase by BCA protein assay. When not in use the columns were stored in a pH 7 50 mM sodium phosphate buffer at 4 °C. A column without protein immobilised, referred to as the dead column was also prepared in order to calculate the dead volume of the column as described by Tessier et al. [59]. The choice of resin for these experiments was based on the highest levels of immobilisation achieved for each protein to be studied.

### 2.3 Self-interaction chromatography

SIC measurements were performed using an Agilent 1100 series liquid chromatograph (Agilent Technologies, Cheshire, UK) consisting of a binary pump, degassex, autosampler, column temperature control unit, Phenomenex Degassex model DG-4400 vacuum four-channel on-line degassex (Phenomenex, Torrance, CA) and two variable wavelength detectors – one before and one after the column. The LC system was controlled and data were collected using Chemstation software version Rev.A.10.02 for LC systems (Agilent Technologies). The protein loaded stationary phase was slurry packed into an empty stainless steel LC column 100 mm long with an i.d. of 4.6 mm (Alltech Associates).

The integrity of the packed columns was confirmed by injecting a 25 μL of a 2% solution of acetone and examining the symmetry of the resulting peak. All experiments were performed on SIC columns with a protein surface coverage of ∼30–40% (i.e. 13.5–17.5 mg/mL for lysozyme) as recommended by Tessier et al. [59]. The impact of varying flow rates, injection concentration and volume on *b*_2_ was assessed for each protein and settings were chosen for the experiments within the range where *b*_2_ was independent of these parameters. All the experiments were carried out at a flow rate of 0.5 mL/min, protein mobile phases contained between 15 mg/mL and 5 mg/mL of protein and the injection size was 10 μL. Injections were repeated in triplicate and the order of solution conditions was programmed randomly to ensure the reliability of the results obtained.

### 2.4 Retention data processing

The retention volume was taken as the first moment of the peak. The chromatographic retention factor (*k*′) was calculated from the retention volume (*V_r_*) as follows:

(2) $$k^{\prime }=\frac{\left(V_{r}-V_{o}\right)}{\left(V_{o}\right)}$$

where *V_o_* is the retention volume in the absence of interactions which was determined using the dead column as described by Tessier et al. [59]. *b*_2_ values were calculated using the following equation:

(3) $$b_{2}=1-\frac{k^{\prime }}{B_{2}^{\mathrm{HS}}\rho_{S}\phi }$$

$B_{2}^{\mathrm{HS}}$ represents the excluded volume or hard sphere contribution calculated from the molecular weight [33]. The total number of immobilised protein molecules per unit area is denoted by *ρ_S_*. The phase ratio *σ* is defined to be *σ* = *A_s_*/*V_o_* which is the total surface available to the mobile phase protein. The phase ratios were interpolated using the work of DePhillips and Lenhoff [17].

### 2.5 Dynamic light scattering

Dynamic laser light scattering (DLS) measurements were performed for protein samples of between 15 and 25 mg/mL (concentration varied in order to achieve the appropriate counts/s range for reliable measurements) with varying pH, ionic strength and salt types using a Beckman Coulter N4 Plus particle sizer instrument with a 10 mW He–Ne laser at 632.8 nm. Triplicate samples were prepared for each condition. The protein and salt solutions were prepared separately and filtered using a 0.22 μm membrane filter prior to analysis. The solutions were then gently mixed in appropriate ratios constituting a 3 mL total solution volume in disposable UV grade plastic cuvettes and quickly inserted into the DLS instrument. All measurements were performed at 25 °C with 5 min of equilibration and automatic time settings for the integration and the intensity correlation function. Sample temperature during measurements was controlled by the built-in Peltier element. Each measurement was taken for 60 s and repeated 10 times over a range of scattering angles. Manufacturer supplied software was used analyse the autocorrelation function with a size distribution profile deconvolution algorithm based on the CONTIN program to generate the intensity-weighted hydrodynamic radius (*R_H_*) distribution of particles in solution. During analysis of the results, any correlation functions with polydispersity values >0.7 were rejected.

## 3 Results and discussion

### 3.1 Comparison of published *B*_22_ data for lysozyme

*B*_22_ data for lysozyme are well reported. However, whilst similar trends are observed in these studies, there is a notable spread in the results reported. Almost all other published SIC work uses peak maximum methodology; however, to obtain more accurate retention time data, good chromatographic analysis practice dictates that first moment methodology should be used in data analysis [15], [42]. Our studies postulate that using first moment methodology gives more meaningful *B*_22_ values that in turn better correlate with observed aggregation propensities, as small differences in retention times due to difference in peak maximum versus centre of mass retention times can result in large differences in *B*_22_ values.

**Fig. 1:**
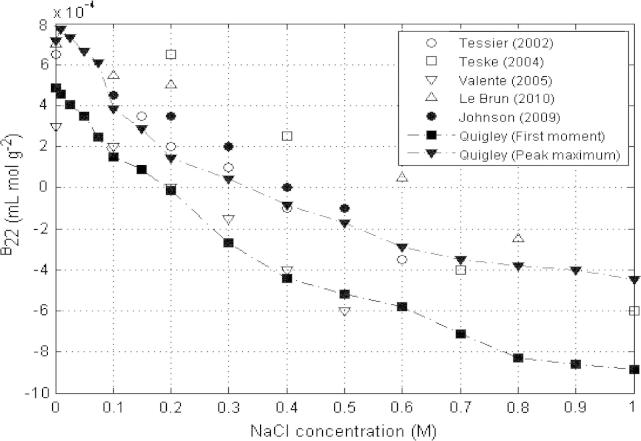
Comparison of peak maximum and first moment *B*_22_ values for lysozyme measured in this work and by 5 other authors under similar buffer conditions (20 mM sodium acetate buffer pH 4.5, 10 μL injection, 0.5 mL/min) with increasing concentrations of NaCl.

Fig. 1 compares reported lysozyme *B*_22_ values from various authors [27], [28], [57], [59], [61] under similar solution conditions (sodium acetate buffer pH 4.5) showing the effect of increasing NaCl concentration. It also displays the difference between the *B*_22_ values obtained in this work using peak maximum and first moment methodologies for calculation of retention times from the same chromatograms. Whilst the results of analysis with peak maximum methodology are in good agreement with results from other authors, using a first moment analysis gives significantly more negative *B*_22_ values but with the same overall trend. At low ionic strengths *B*_22_ values were positive but a change in sign was observed at a NaCl concentration of approximately 0.20 M NaCl when using first moment methodology for calculation of *B*_22_. Using peak maximum analysis *B*_22_ remained positive until a concentration of approximately 0.35 M NaCl in this investigation, whilst on average *B*_22_ values in the literature (Fig. 1) could be approximated to become negative at about 0.40 M. This work suggests that using the peak maximum is an underestimate of retention time that results in an overestimate of *B*_22_, consistent with the observed discrepancies. The difference between the *B*_22_ values calculated using first moment and peak maximum methodologies becomes increasingly large with increasing ionic concentration due to increased peak asymmetry or tailing observed in chromatograms under these conditions, an effect that is observed in other studies [59].

#### 3.2 Ionic strength and type

Salts have a complex effect on protein physical stability as they modify solubility, conformational stability and the rate of aggregate formation. The net effect of a given salt on protein–protein interactions is a balance of the multiple mechanisms by which that salt can interact with protein molecules. In both industrial applications and scientific research salts are universally used in protein solutions to control ionic strength, pH and osmolality which moderate protein solution behaviours such as protein aggregation, crystallisation and precipitation. In the present work, the *b*_2_ measurements presented are intended to provide an inclusive view of the different effects of sodium salt systems on protein–protein interactions and in particular protein stability. For this reason *b*_2_ was determined over a range of ionic strengths for lactoferrin, catalase and con A (at pH 7) and lysozyme (pH 4.5) with different salts. The four proteins were selected to have different isoelectric points and molecular weights in order to capture a number of different protein–protein interaction phenomena.

**Fig. 2:**
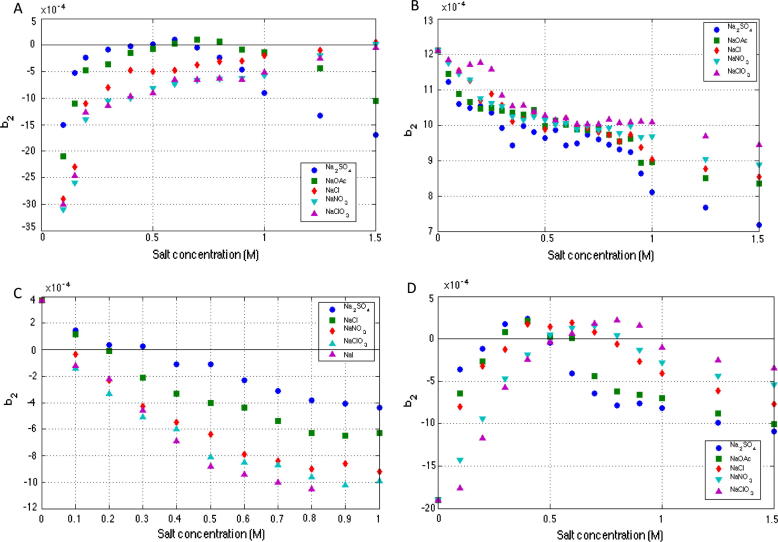
The reduced osmotic virial coefficient (*b*_2_) for catalase (A), lactoferrin (B), lysozyme (C) and con A (D) (measured by SIC) as a function of increasing ionic strength and type (20 mM sodium phosphate buffer pH 7.0 for rhLf, catalase and con A, and 50 mM sodium acetate buffer pH 4.5 for lysozyme, 0.5 mL/min, 10 μL injections).

Fig. 2 illustrates that the effect of the ionic strength and salt type in a solution is very much protein dependent. For reasons of clarity, error bars are not shown for *b*_2_ values but are typically close in size to the data point symbols used. Lysozyme *b*_2_ values (Fig. 2C) decrease with increasing ionic strength, indicating that attractive interactions are becoming increasingly dominant. This trend has been widely reported and although *b*_2_ does not provide any information on the physical origins of the experimentally observed interaction patterns, this trend is commonly interpreted as resulting from increased screening of the protein’s surface charges with increasing ionic strength, a classic electrical double layer effect [44], [43]. It appears as though this trend is somewhat unique, with most other proteins exhibiting very different interaction behaviour, possibly reflecting more complex protein structures and chemistry for these other larger proteins [19]. Indeed, lysozyme has a history of displaying atypical protein interaction behaviour and was the first protein demonstrated to follow an inverse Hofmeister series of reactivity in salt solutions at a pH below the pI of the protein; a trend which can be seen in the measured *b*_2_ values in Fig. 2C [45], [65], [9]. This reversal has since been shown to apply not only to lysozyme but also to a range of other small proteins including α-crystallins, ATCase and BMV [20]. The SIC data for lactoferrin, catalase and con A indicate that the greatest changes in *b*_2_ values occurred with the addition of salts with more strongly hydrated anions (SO^2−^ and Cl^−^) in the given range of concentrations examined. Indeed, the *b*_2_ trend for lactoferrin shows that this protein also follows an inverse Hofmeister series at this pH, which is not unexpected as lactoferrin is positively charged under these conditions. The SIC data for con A and catalase are more complex. Both proteins are negatively charged at pH 7 and the investigations demonstrated that below salt concentrations of around 0.5–0.8 M these proteins follow a direct Hofmeister series trend. At higher salt concentrations these proteins undergo a reversal and these proteins then follow the inverse Hofmeister series. The exact physical origin of the inverse and direct Hofmeister series still remains challenging area of study but the effect is thought to stem from an interplay of ionic sizes, hydration phenomena and dispersion forces as well as the specific chemical and physical properties of the peptides and proteins themselves [50], [9], [39].

At low ionic strengths *b*_2_ values for catalase (Fig. 2A) and con A (Fig. 2D) are very negative indicating that protein–protein interactions would be expected to be strongly attractive, and that these two proteins are unstable under these solution conditions. Since there are currently no validated computational molecular models able to fully describe the effects of salt on protein–protein interactions it can be difficult to identify with certainty the physical origin of these trends. However, such trends have previously been observed under certain solution conditions for ribonuclease A, α-chymotrypsinogen, β-lactoglobulin A, catalase and certain monoclonal antibodies [63], [40], [37], [58], [19], [54] and this behaviour is thought to be the result of complementary electrostatic interactions between patches of oppositely charged residues on the protein’s surface [14], [58], [54]. This theory is supported by two aspects of the *b*_2_ trend; firstly that attractive interactions are screened at lower salt concentrations for salts higher up the Hofmeister series of reactivity and secondly that it virtually vanishes above 0.25 M salt concentration. This observation is consistent with the characteristics of an electrostatic phenomenon as described by the Debye–Hückel theory [25], [24]. Furthermore, this explanation has been shown to agree with computational studies that demonstrate specific pairwise electrostatic interactions can be attractive in individual configurations [34], [30], [21].

Increasing salt concentration in solutions of catalase and con A resulted in *b*_2_ values increasing up to about 0.50 M as these complimentary electrostatic charges are increasingly screened, resulting in decreased protein–protein attractive interactions. For catalase *b*_2_ values almost plateaued between 0.50 M and 1.50 M in solutions of NaCl, NaNO_3_ and NaClO_3_ whilst con A *b*_2_ values decreased once more above 1.0 M indicating improved stability for both proteins even at very high concentrations. In fact, *b*_2_ values for catalase in these salt solutions were highest at 1.50 M and whilst some level of stability at high concentrations is not unexpected for these weakly hydrated salts, it is surprising that catalase should be most stable at such high salt concentrations. This pattern of interactions demonstrates that proteins can be stable in solution when electrostatic interactions are screened, and as such is at odds with the classical DLVO theory of protein stability. However, in the presence of Na_2_SO_4_ and NaOAc for catalase and all salts studied for con A, higher salt concentrations (above approximately 0.7–0.8 M) resulted in decreased *b*_2_ values indicating a return to typical DLVO theory predicted behaviour, with increased screening of the proteins surface charges with increasing ionic strength, a classic electrical double layer effect [44], [43].

In Fig. 2B lactoferrin *b*_2_ values remain strongly positive for all salts and all salt concentrations indicating that protein–protein interactions are repulsive under all conditions. This suggests that lactoferrin has a quite remarkable level of stability and raises some seminal questions as to what confers such incredible stability to this specific protein molecule. Lactoferrin is a multi-functional iron-binding glycoprotein from the transferrin family. It has been shown that the thermal stability of lactoferrin is dependent on the binding of Fe with reduced levels of bound Fe at reduced pH resulting in decreased *T_m_* values [26], [53]. Other studies also indicate that glycosylation of lactoferrin also plays a role, with the thermal stability of the protein being influenced by the characteristics of the specific glycans present [52], [62]. Recent studies on bovine lactoferrin have highlighted its unusual solution properties such as the charge asymmetry of the lactoferrin molecule as well as the fact that it carries a net positive charge at physiological pH (pI ∼ 8.0–8.5) [31]. This study did however report on the presence of 100 nm aggregates which formed at 0.1 M NaCl, which contrasts markedly with our current study, and raises questions about potential differences in the solution behaviour of bovine lactoferrin and the recombinant human lactoferrin studied here.

**Fig. 3:**
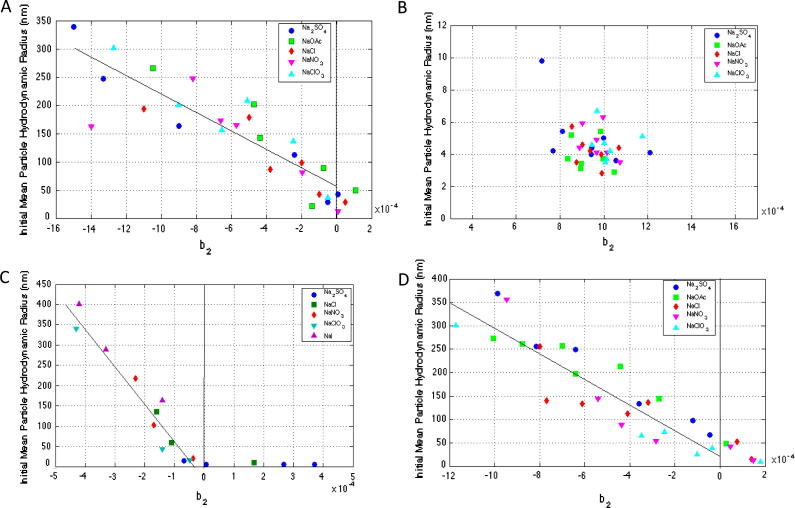
Correlation between SIC measured *b*_2_ values and initial hydrodynamic radius of catalase (A), lactoferrin (B), lysozyme (C) and concanavalin A (D) measured in salt solutions of varying concentration and ion type (20 mM sodium phosphate buffer pH 7.0 for lactoferrin, catalase and concanavalin A, and 50 mM sodium acetate buffer pH 4.5 for lysozyme).

In order to determine at what concentration protein aggregation is induced by the varying salt solution conditions, the protein hydrodynamic radius was tracked using DLS measured particle size under identical buffer conditions (although not all conditions were replicated as at some of the higher ionic strengths aggregation proceeded too rapidly to accurately measure using DLS). At around pH < ∼5.0 lysozyme has been reported to exist in monomeric form where its hydrodynamic radius can be expected to be approximately 1.9 nm [36]. A single recombinant lactoferrin monomer can be expected to be roughly 4 nm [4], a catalase tetramer has a hydrodynamic radius of 5.2 nm [51], and a con A tetramer has a hydrodynamic radius of 4.3 nm [2]. In this study initial particle hydrodynamic radius is reported at typically *t* = 10 min and particle size is used here as a general measure of protein aggregation behaviour with particles of 2× or greater radii than the stated hydrodynamic radii for the protein were presumed to indicate the presence of some level of protein aggregation. It would therefore be predicted that negative *b*_2_ values should correlate with this increased particle size. However, protein aggregation can of course be driven by vastly different mechanisms and further analysis would be required to ascertain whether this eventually leads to amorphous sub-visible aggregation, liquid–liquid phase separation, precipitation, and gelation or crystallisation phenomena. The results are shown in Fig. 3 correlated against *b*_2_ values over a range of ionic strengths of salts between 0.10 M and 1.00 M. For reasons of clarity error bars are not included in DLS data but typical variations are around 10–20% of measured particle size.

Considering the *B*_22_ data for lysozyme with NaCl published by other workers as well as the data from this study analysed using the peak maximum method (Fig. 1), it is clear that *B*_22_ values in almost all cases do not become negative until concentrations of typically 0.40 M or greater in NaCl. However, DLS data (Fig. 3C) show that at just half this concentration, 0.2 M NaCl, the onset of aggregation can be clearly observed with particle radius of ∼50 nm. However, retention times analysed using first moment methodology show that *b*_2_/*B*_22_ passes through 0 at 0.2 M NaCl and clearly correlate much better with the aggregation behaviour of lysozyme for these solutions. Light scattering studies reported also support this first moment methodology. Rosenbaum and Zukoski [48] reported that *B*_22_ at a pH of 4.6 values becomes negative at 0.2 M NaCl, whereas Velev et al. [63] reported for pH 4.5 *B*_22_ values changed from positive to negative values at 0.2 M NaCl. Piazza and Pierno [38] also reported virial coefficients that transitioned from positive to negative values between 0.2 M and 0.3 M NaCl.

Broadly speaking a good correlation can also be observed between *b*_2_ values of all four proteins and aggregation propensity examined using DLS of all ionic solution conditions investigated. The greatest increase in the measured particle size of aggregates corresponds to those with the most negative *b*_2_ values, suggesting that these conditions correspond to the fastest rates of aggregation; the same is also true conversely for the salt conditions under which the proteins remained most stable.

**Fig. 4:**
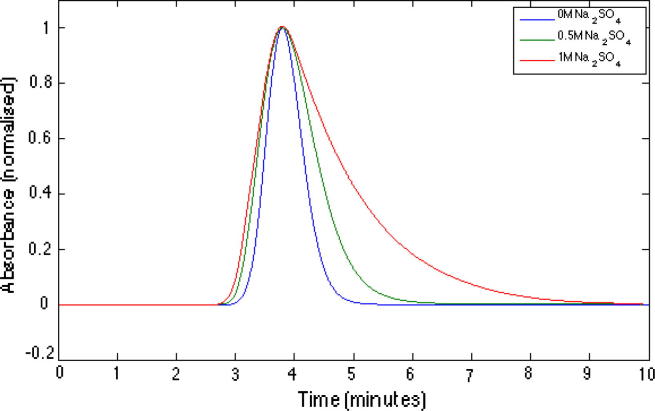
Example of lactoferrin retention peaks increased tailing with increasing concentration of sodium sulphate (20 mM sodium phosphate buffer, pH 7, 0.5 mL/min, 10 μL injections). The peaks have been shifted to have the same peak maximum and the absorbance scale normalised for better comparison of peak shape.

However, time resolved aggregation studies of lactoferrin revealed that at sodium sulphate concentrations of above 0.70 M the formation of ∼200 nm (hydrodynamic radius) aggregates was observed within a period of 0.5–24 h. As such *b*_2_ values do not properly describe aggregation propensity of lactoferrin under these conditions. It is likely that high concentrations of sodium sulphate result in an unfolded or partially unfolded lactoferrin conformation that is the aggregation prone species and that in the case of this aggregation mechanism the conformational change rather than colloidal interactions is the rate limiting step for this reaction. As *b*_2_ measures overall protein–protein interactions it is effectively a weighted result from all protein species present. As such, under conditions where the predominant species is the native protein small changes in the population of the unfolded or partially unfolded protein do not contribute significantly towards the net interaction value given by *b*_2_. Similar findings have been reported for other aggregation cases in the literature [6]. Interestingly, at high sodium sulphate concentration significant peak tailing is observable on SIC chromatograms (Fig. 4). Thus, a detailed analysis of SIC peak shape and the spread of *b*_2_ values as discussed in previous work [42] could provide indications of longer term aggregation trends that may be useful for assessing stability of protein formulations for long term storage.

### 3.3 pH

**Fig. 5:**
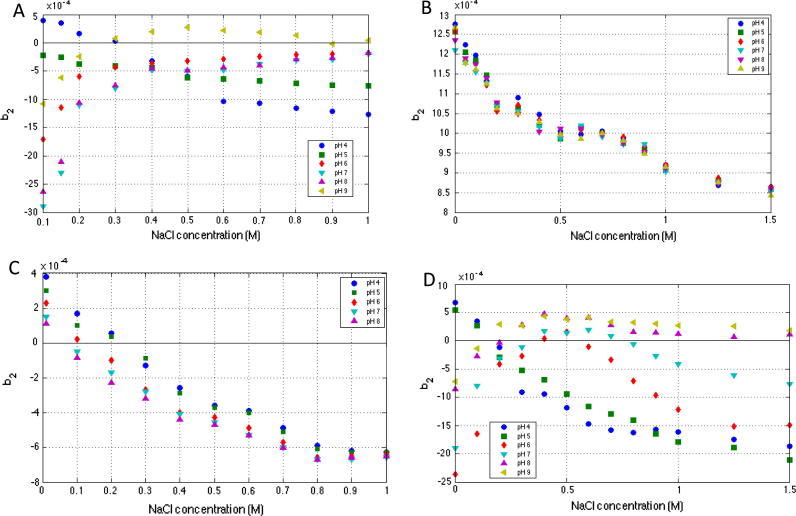
*b*_2_ for catalase (A), lactoferrin (B), lysozyme (C) and Con A (D) as a function of pH (20 mM sodium phosphate and 20 mM sodium acetate buffers, 0.5 mL/min, 10 μL injections) with increasing NaCl concentration.

Solution pH determines the charge distribution in a protein, modifies the magnitude and geometry of ionic interactions, alters the protein hydration state, and has also been shown to induce conformational changes [55], [41], [56]. As such changes in pH can have a significant impact on protein–protein interactions. Fig. 5 shows *b*_2_ values for catalase, lactoferrin, lysozyme and con A respectively in solutions of NaCl as a function of pH. At low salt concentrations of below 0.30 M NaCl, catalase and con A *b*_2_ values (Fig. 5A and D) indicate attractive protein–protein interactions, with the exception of pH 4.0 (and pH 5.0 for con A) under which protein–protein interactions are repulsive and decrease with increasing ionic strength at these pHs. Between pH 6.0 and 9.0 for catalase and pH 7.0 and 9.0 for con A *b*_2_ values rise with increasing NaCl concentration before plateauing. Measured *b*_2_ values for con A at pH 7.0 and 6.0 then begin to decrease at NaCl concentrations above ∼0.70 M. It is important to note that con A exists in its tetrameric form at pH 7 and above but below pH 6 it exists as a dimer. As such catalase and con A stability would be expected to be highest at high pH and medium ionic concentrations or low pH and low salt concentrations.

Solution pH appears to have very little effect on lactoferrin–lactoferrin interactions. There is no discernable difference in *b*_2_ trend as pH is varied (Fig. 5B). It has been shown previously that lactoferrin uniquely retains bound iron over a broad pH range and this unique iron binding stability has been found to be imparted primarily by the carbonyl-terminal domain which functions cooperatively to stabilise the iron-binding to the amino-terminal domain [1]. This binding is perhaps a contributing factor in overall high stability of lactoferrin. Fig. 5C shows that for lysozyme increasing pH results in increasingly negative *b*_2_ values and that the effect of NaCl on *b*_2_ values at higher pH becomes reduced as the pH approaches the pI of lysozyme; pH 11.0. At a pH of 11.0 lysozyme would exhibit an overall neutral charge and therefore increasing the pH progressively reduces the surface charge of lysozyme and as a result the electrostatic stabilising effects are reduced.

Both catalase and con A display repulsive protein–protein interactions at pH below their pI that become predominantly attractive at high ionic strengths due to increasingly screened repulsive electrostatic interactions leading to short range attractive interactions including those caused by the reduced hydration of the proteins at low pH. At high pH (above their pI) the opposite dependence on ionic strength is true. The similarity in behaviour of catalase and con A to ribonuclease A, α-chymotrypsinogen and certain mAbs is once again evident and again potentially due to the heterogeneous distribution of surface charge observed for each protein [14], [58], [19], [64], [54]. This is once again very different to the behaviour observed for lysozyme [63], [48]. Lactoferrin pH behaviour is different again but could be better compared to interactions of myoglobin and bovine serum albumin which display repulsive protein–protein interactions over a range of pH values, which are diminished somewhat at high ionic strengths but not sufficiently to induce attractive protein–protein interactions [5], [59].

**Fig. 6:**
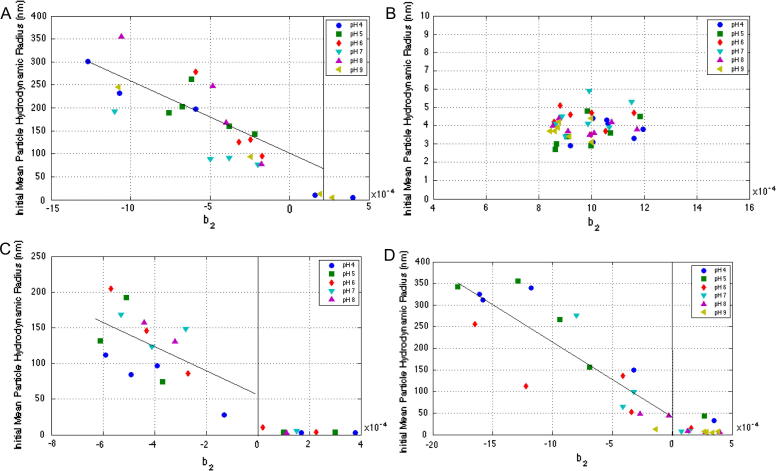
Correlation between SIC measured *b*_2_ values and initial hydrodynamic radius of catalase (A), lactoferrin (B), lysozyme (C) and concanavalin A (D) measured in solutions of varying pH (20 mM phosphate and sodium acetate buffers pH 4–9) over a range of NaCl concentrations between 0.01 and 1 M.

Fig. 6 shows the correlation between *b*_2_ and initial hydrodynamic radius for all four proteins at various pH. The agreement between the results is very good, with almost no self-associative behaviour being observed for positive virial coefficient values, and that which does occurs mostly at the dimer level. Furthermore, growth in initial hydrodynamic radii increases in line with increasingly negative *b*_2_ values.

## 4 Conclusion

The present measurements are amongst the most extensive sets of *b*_2_ values reported, describing the effects of salt type, salt concentration and pH on protein–protein interactions for catalase, lactoferrin, lysozyme and concanavalin A. Use of first moment analysis of self-interaction chromatographic peaks is shown to provide more accurate *b*_2_ values and this is reflected by improved correlation with observed protein aggregation behaviour. Comparisons between measured *b*_2_ values and measured protein aggregate sizes gave good linear correlations for a range of systems, and *b*_2_ values of <0 × 10^−4^ mL mol g^−2^ were found to be a good predictor of protein aggregation propensity. It is also noteworthy that lactoferrin *b*_2_ values remained strongly positive (a sign of repulsive protein–protein interactions) for all conditions tested indicating that this is a remarkably stable protein under a wide range of typically unfavourable solution conditions. Furthermore, it has been demonstrated that *b*_2_ measurements can be used to predict the aggregation stability of proteins and for screening studies to identify solution conditions that minimise protein aggregation.
